# Modeling rejection immunity

**DOI:** 10.1186/1742-4682-9-18

**Published:** 2012-05-20

**Authors:** Andrea De Gaetano, Alice Matone, Annamaria Agnes, Pasquale Palumbo, Francesco Ria, Sabina Magalini

**Affiliations:** 1 CNR-IASI BioMatLab, UCSC Largo A. Gemelli 8, 00168, Rome, Italy; 2 Department of Surgery-Transplantation Service Catholic University, A. Gemelli Hospital, Rome, Italy; 3 Institute of General Pathology Catholic University, Largo F. Vito 1, 00168, Rome, Italy

## Abstract

**Background:**

Transplantation is often the only way to treat a number of diseases leading to organ failure. To overcome rejection towards the transplanted organ (graft), immunosuppression therapies are used, which have considerable side-effects and expose patients to opportunistic infections. The development of a model to complement the physician’s experience in specifying therapeutic regimens is therefore desirable. The present work proposes an Ordinary Differential Equations model accounting for immune cell proliferation in response to the sudden entry of graft antigens, through different activation mechanisms. The model considers the effect of a single immunosuppressive medication (*e.g.* cyclosporine), subject to first-order linear kinetics and acting by modifying, in a saturable concentration-dependent fashion, the proliferation coefficient. The latter has been determined experimentally. All other model parameter values have been set so as to reproduce reported state variable time-courses, and to maintain consistency with one another and with the experimentally derived proliferation coefficient.

**Results:**

The proposed model substantially simplifies the chain of events potentially leading to organ rejection. It is however able to simulate quantitatively the time course of graft-related antigen and competent immunoreactive cell populations, showing the long-term alternative outcomes of rejection, tolerance or tolerance at a reduced functional tissue mass. In particular, the model shows that it may be difficult to attain tolerance at full tissue mass with acceptably low doses of a single immunosuppressant, in accord with clinical experience.

**Conclusions:**

The introduced model is mathematically consistent with known physiology and can reproduce variations in immune status and allograft survival after transplantation. The model can be adapted to represent different therapeutic schemes and may offer useful indications for the optimization of therapy protocols in the transplanted patient.

## Background

It is unfortunately not rare in medical practice that some diseases lead to organ failure, which may eventually require organ transplantation. The liver, the kidney and the heart are the most frequently transplanted organs. Diseases leading to organ transplantation span a wide spectrum of medical conditions: cancer, infections, autoimmune and degenerative diseases. Transplantation into the recipient of a foreign organ (graft), even from an individual of the same species (allograft), if left to itself causes rejection, a strong response by the recipient’s immune system leading to irreversible damage of the graft. Depending on the time-frame over which rejection occurs, “acute” rejections are differentiated from “chronic” ones. Acute rejection develops in the first few weeks or months after transplantation and is produced by cellular and molecular mechanisms, which may partially differ from those leading, over the course of many months or years, to chronic rejection.

After many decades of experimentation on animals and cells, and of the development of pharmacological tools, organ transplantation has evolved into a common therapeutic procedure. The success of a solid organ transplant relies in equal measure on the technical aspects of the implant and on the recipient’s acceptance or tolerance of the implanted graft. This last phenomenon is clinically induced by the administration of immunosuppressive drugs, which specifically decrease the recipient’s reactivity towards the graft, thus allowing the maintenance of the functional activity of the organ. Currently available drugs belong to several classes (calcineurine inhibitors (CNI), antimetabolites, target of rapamycin (TOR) inhibitors, steroids, and monoclonal antibodies) [1]. Canonical combinations of these drugs are typically used by the attending physician in a rather standardized fashion, attempting to maintain measurable drug plasma concentrations within established limits. Episodic and emergency use of immunosuppressive agents is then performed if signs of rejection become clinically evident.

Acute rejection response has been extensively studied *in vivo* and *in vitro*. From available studies, indications on the action of several drugs in controlling acute rejection and maintaining vitality of the graft have been obtained. However, even if the recipient initially accepts the graft, the more insidious and slow phenomenon of chronic rejection often ensues. The pathogenesis of chronic rejection is less well known and may depend not only on a partially different set of immune response mechanisms, but also on actual drug toxicity, recommending the use of the minimal clinically effective dose of medication.

Rejection has been widely described from a medical and biological viewpoint, but there have so far been no mathematical models describing this process. Mathematical models have been used to describe immunological behavior for a long time, beginning with the classical SIR model (Susceptible-Infectious-Recovered) first created for investigating the progress of an epidemic [2]. Several models describing the immune response in a number of diseases exist (HIV infection, tuberculosis, tumors..) [3-7] but, at present, only one model representing immune system dynamics during transplantation [8] has been published, which has the important aim to investigate T-cell population growth mechanisms, using thymus transplantation to follow the development of T-cells and their regulatory signals. The goal of the present work is somewhat different, in that we attempt to describe the main features of the immune system dynamics during general solid organ rejection. This will allow in the future the description of possible consequences of different therapeutic regimens. From the immunologic viewpoint, rejection mechanisms are substantially different from other immune responses (*e.g.* towards HIV, tuberculosis, or tumors), and the development of a specific model seems therefore warranted. Such a model should help transplantation clinicians and allied health care personnel in forecasting and treating rejection, without relying solely on empirical protocols. A good mathematical model should eventually allow the physician to consider in real time the several interrelated aspects of the immune response to transplantation, while jointly incorporating the known pharmacokinetics of the many potentially useful available drugs. The ultimate goal would be to help bridge the gap between the pharmacology and the biology of transplantation, explaining or at least representing the temporal relations between drug efficacy, possible drug adverse effects and the development of immune tolerance or graft acceptance.

In the present work we propose a tentative mathematical model of the rejection towards a solid organ transplant (kidney, liver, pancreas, heart). This model describes the evolution of the main cellular immune response as well as the kinetics and action of a single representative drug (e.g. cyclosporine). In order to have some physiological support for parameter assessment, a Mixed Lymphocyte Reaction (MLR) experiment was performed, on the basis of which the clonal expansion rate of T-cells could be determined. This experiment was chosen because the parameter of interest for the model was directly computable from the experimental data. Simulations with MatlabⒸ2010b have been performed and, on the basis of the experimentally determined clonal expansion rate and of relevant published material, the other model parameters were calibrated. While the current model is relatively simple, it introduces the main elements needed for the eventual description of more detailed response dynamics. Three case scenarios are discussed: non-immunosuppressed transplantation and two immunosuppressive therapeutical regimens, one with moderate drug dose and the other with high drug dose. Additional simulations were made in order to explore different hypothetical therapy scenarios. Also, three state variables were selected as being most clinically relevant, and a sensitivity analysis was performed on these at two time points (one and ten years post-transplant).

## Methods

### Relevant physiology

After transplantation, the majority of exogenous molecules on the allograft are recognized by the immune system as self-antigens, as they are the same in both the donor and recipient. The main donor molecules which induce the immune response are those coded from the Major Histocompatibility Complex (MHC) genomic locus, which is the most polymorphic one on the human genome (it is essentially impossible to find two persons with the same gene cluster, except for monozygotic twins). Proteins coded by the MHC are located on the cell surface and bind antigen epitopes, creating a complex, which is recognized by the T-Cell Receptor (TCR) on T-lymphocytes, inducing the immune response. Cells which carry the MHC/epitope complexes are called Antigen Presenting Cells (APCs) [9].

**Figure 1:**
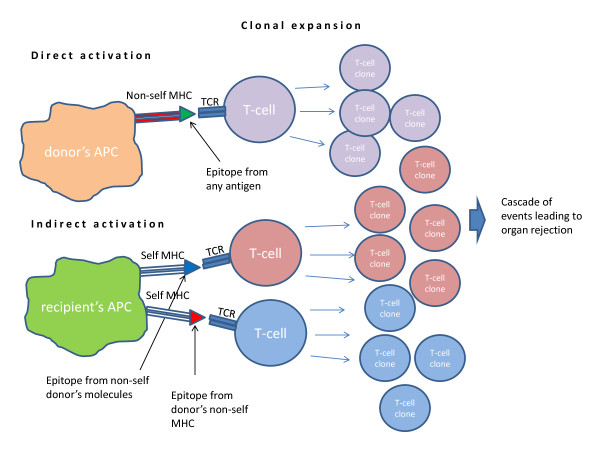
**Direct and indirect activation mechanisms.** With the direct activation mechanism the donor’s Antigen Presenting Cell, expressing its MHC molecule (red) and carrying a general epitope (green), is recognized from the T-Cell Receptor and activates T-cell clonal expansion. In this case it is the actual MHC molecule which is recognized as non-self from the TCR. In the lower part of the figure, the two possible indirect activation scenarios: self APC, expressing self MHCs, can present an epitope from graft antigens recognized as non-self (blue) or from donor’s MHC (red).

In the conventional immune response, self-APCs carry external antigen epitopes bound to self-MHC molecules. APCs normally circulate in the body and are found in every organ - during transplantation many APCs from the donor are introduced in the recipient, and they carry the donor’s MHCs. In fact, MHCs on donor cells are the major target of the rejection immune response [10], as T-cells can recognize complexes formed by allogenic MHCs [11]. This mechanism is called “direct activation”, since there is no processing of the antigen, and it is faster than the indirect activation, where recipient’s APCs have to process donor’s antigen [12]. In Figure 1 a schematic representation of the direct and indirect mechanisms is shown.

Every different MHC on the surface of an APC can be recognized by and activate a different T-cell clone to proliferate (“clonal expansion”). It should be noticed that in the indirect mechanism many of the foreign epitopes involved are donor MHC fragments, due to the above mentioned polymorphism [13]. However, indirect activation can also occur in response to peptides derived from other molecules present in the allograft. “Alloreactive T-lymphocytes are *[in any case]* requisite mediators of allograft rejection” [11].

At the level of detail used in the present work, CD4+ and CD8+ T-lymphocytes (“helper” and “cytotoxic”) are not distinguished, and we consider them together as T-cells mediating rejection. The reasons for this simplification are that both T-cell types increase in number during the immune response and that they can both be activated by exogenous antigens. It has recently been shown that both class I and II MHC molecules (respectively recognized by CD4+ and CD8+ T-cells) can bind extracellular antigens (“cross priming”), while it was previously thought that class I MHCs could only present intracellular antigen [14]. In the course of direct activation, APCs are either destroyed or will eventually undergo apoptosis. Since donor’s APCs do not reproduce in the allograft, direct T-cell activation is a time-limited process. The immune response, however, does not terminate since there is continuous supply of donor-specific molecules, produced from the constantly proliferating cells in the graft. T-lymphocyte activation occurs as long as the graft is present in the recipient.

We can thus appreciate two main phases of the immune response to the graft: an initial strong response, sustained by the activation of T-cells due to a large but fading quantity of donor APCs, necessitating aggressive immune suppression therapy; and a later, more or less constant indirect activation of T-cells, sustained by continuously produced graft epitopes, for which less aggressive therapy is sufficient. It has in fact been reported that the number of directly activated T-cells is larger than the number of indirectly-activated T-cells, the latter constituting less than 10 percent of the total cellular alloimmune repertoire [15].

Two aspects of graft rejection have not been explicitly included in the model for simplicity. The first is the increased production of lymphocytes from lymphoid organs, which receive various signals from stimulating molecules (cytokines). This mechanism has been shown [16] to take place when an inflammatory process (*i.e.* rejection) is ongoing. The second aspect of rejection is the appearance of the graft-*versus*-host disease, where donor’s T-cells present in the allograft react towards recipient’s antigens. This last phenomenon depends on the type of the transplanted organ and may be negligible in most cases of solid organ transplantation.

MLR is an *in vitro* experiment used to study alloreactive T-cells response to exogenous MHC molecules: it is used in clinical practice as a prediction rejection test before performing organ transplantation. MLR is induced growing mononuclear cells of an individual with those of another individual, these cells being isolated from peripheral blood: the difference between MHC *loci* of the two individuals induces clonal expansion of alloreactive lymphocytes [17]. One of the model parameters, the one corresponding to the clonal expansion rate of T-cells, was determined by an MLR experiment.

In the present model we consider one of the most frequently used immunosuppression therapy protocols, based on calcineurine inhibitors (*e.g.* cyclosporine) [1], which block T-cell clonal expansion. Drugs of this class inhibit signal transduction when TCRs recognize the epitope, so that the cell does not proliferate even when activated. Other types of therapy, acting through different mechanisms, could also be considered, extending the present approach.

### The model

**Figure 2:**
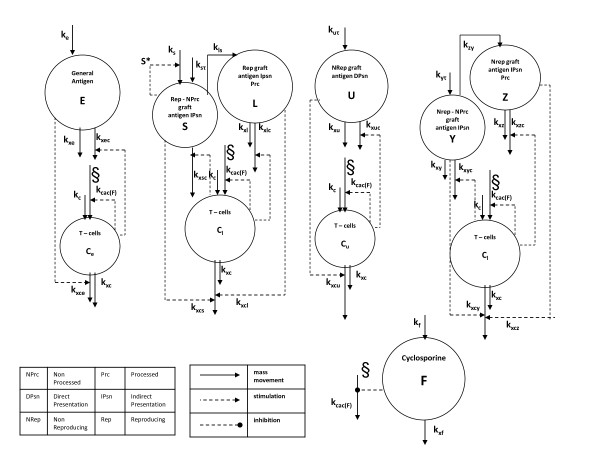
**Model block diagram.** State variables are represented with circles, the above ones for the different antigen types, the bottom ones for specific T-cells. On the bottom right the pharmaceutical dynamics is represented, the symbol *§* indicates the site of action of the drug. Solid arrows represent mass transfers, while dashed lines indicate stimulation and inhibition (arrow end and dot end, respectively).

The model is represented in block diagram form in Figure 2, showing all compartments and the relative dynamics. Organ transplantation is assumed to occur at time *t*_*τ*_ in the life of the patient. Before transplantation, the major components of the immune system involved in transplant rejection are assumed to be at equilibrium. The choice of the time *t*_0_, at which simulations begin, is therefore irrelevant, as long as *t*_0_ < *t*_*τ*_. In order to represent organ damage, we assume that antigen is released into the blood stream proportionally with the viable mass of the corresponding tissue, and consequently that the viable graft mass is proportional to bloodstream antigen mass, so that a substantial decrease in antigen concentration will indicate organ failure.

With the transplantation of an allograft at time *t* = *t*_*τ*_, the state of the immune system is suddenly and dramatically altered. The entry of a large amount of already processed foreign antigen, and the continuous production of foreign antigen by the functional graft tissue, induce a strong response by the host’s immune system. If this response is not controlled by immunosuppression, rejection and loss of the organ follow. Depending on the time and mechanisms needed to activate the immune response and on the duration of antigen permanence in the body, we describe different antigen types and assume that a specific T-cell type corresponds to each antigen type. In our model we use nonlinear ordinary differential equations to describe antigen and T-cell dynamics, using appropriate coefficients to specify the effect of antigens on corresponding T-cells and viceversa. State variables are generally defined in terms of concentrations, and we suppose a single vast plasma/interstitial fluid volume space where, given time, all species distribute. State variables and parameters, with corresponding units of measurement, are listed in Tables 1 and 2, respectively.

**Table 1:** Model variables

**Variables**	**Units**	**Description**
*t*	years	time
*E*	*μ*g/l	generic antigen (antigenically active molecules): complexes MHC-antigenic epitopes
*S*	*μ*g/l	antigen allograft cells, which reproduce in time, non-processed form(indirect presentation)
*L*	*μ*g/l	antigen allograft cells, which reproduce in time, processed form (indirect presentation)
*U*	*μ*g/l	antigen from APCs in the allograft, not reproducing in it (direct presentation)
*Y*	*μ*g/l	antigen from APCs in the allograft, not reproducing in it, non-processed form (indirect presentation)
*Z*	*μ*g/l	antigen from APCs in the allograft, not reproducing in it, processed form (indirect presentation)
*C*_*e*_	cell/*μ*l×10^3^	antigen *E* specific T-lymphocytes
*C*_*l*_	cell/*μ*l×10^3^	antigen *L* specific T-lymphocytes
*C*_*u*_	cell/*μ*l×10^3^	antigen *U* specific T-lymphocytes
*C*_*z*_	cell/*μ*l×10^3^	antigen *Z* specific T-lymphocytes
*F*	ng/ml	cyclosporine
*k*_*cac*_(*F*)	l/*μ*g/year	T-cells production rate (T-cells clonal expansion), antigen and lymphocyte dependent, as function of *F*
*k*_*sτ*_	*μ*g/l	*S* entry rate at time *t*_*τ*_ (from reproducing cells, to be processed)
*k*_*uτ*_	*μ*g/l	*U* entry rate at time *t*_*τ*_ (from non-reproducing cells, directly presented)
*k*_*yτ*_	*μ*g/l	*Y* entry rate at time *t*_*τ*_ (from non-reproducing cells, to be processed)

**Table 2:** Model parameters

**Paramaters**	**Value**	**Units**	**Description**
*t*_0_	35	years	minimum time considered by system, in days
*t*_*τ*_	40	years	time in which transplantation occurs, in days
*k*_*e*_	1	*μ*g/l/year	constant environment antigen entry into the body
*k*_*xec*_	1.5	*μ*l/cell/year×10^−3^	second order *C* dependent *E* elimination rate constant
*k*_*xsc*_	0.15	*μ*l/cell/year×10^−3^	second order *C* dependent *S* elimination rate constant
*k*_*xlc*_	0.7	*μ*l/cell/year×10^−3^	second order *C* dependent *L* elimination rate constant
*k*_*xuc*_	0.5	*μ*l/cell/year×10^−3^	second order *C* dependent *U* elimination rate constant
*k*_*xyc*_	0.5	*μ*l/cell/year×10^−3^	second order *C* dependent *Y* elimination rate constant
*k*_*xzc*_	1	*μ*l/cell/year×10^−3^	second order *C* dependent *Z* elimination rate constant
*k*_*xe*_	1	year^−1^	first order elimination rate from compartment *E*
*k*_*xl*_	0.7	year^−1^	first order elimination rate from compartment *L*
*k*_*xu*_	0.001	year^−1^	first order elimination rate from compartment *U*
*k*_*xy*_	0.001	year^−1^	first order elimination rate from compartment *Y*
*k*_*xz*_	1	year^−1^	first order elimination rate from compartment *Z*
*k*_*ls*_	1.5	year^−1^	first order transfer rate from compartment *S* to *L*
*k*_*zy*_	6	year^−1^	first order transfer rate from compartment *Y* to *Z*
*k*_*xc*_	1	year^−1^	first order elimination rate from compartment *C*
*k*_*xce*_	1	l/*μ*g/year	second order *E* dependent *C*_*e*_ elimination rate constant
*k*_*xcs*_	0.02	l/*μ*g/year	second order *S* dependent *C*_*l*_ elimination rate constant
*k*_*xcl*_	0.02	l/*μ*g/year	second order *L* dependent *C*_*l*_ elimination rate constant
*k*_*xcu*_	0.02	l/*μ*g/year	second order *U* dependent *C*_*u*_ elimination rate constant
*k*_*xcy*_	0.02	l/*μ*g/year	second order *Y* dependent *C*_*z*_ elimination rate constant
*k*_*xcz*_	0.02	l/*μ*g/year	second order Z dependent *C*_*z*_ elimination rate constant
*k*_*c*_	0.3	cell/*μ*l/year×10^3^	basal lymphocyte production
*k*_*s*_	2	1/year	first order S dependent S production and elimination rate
			constant
*S*^∗^	7	*μ*g/l	allograft maximum regeneration rate
*κf*	0	ng/ml/year	drug delivery rate - scenario 1 (no drug treatment)
*κf*	36500	ng/ml/year	drug delivery rate - scenario 2 (moderate drug treatment)
*κf*	127750	ng/ml/year	drug delivery rate - scenario 3 (high drug treatment)
*k*_*xf*_	365	1/year	first order elimination rate from compartment F
*k*_*cacF*_	2.92	l/*μ*g/year	T-cells production rate (T-cells clonal expansion), A and c
			dependent
*S*_*τ*_	1000	*μ*g/l	antigen S concentration at time *t*_*τ*_
*U*_*τ*_	1000	*μ*g/l	antigen U concentration at time *t*_*τ*_
*Y*_*τ*_	1000	*μ*g/l	antigen Y concentration at time *t*_*τ*_
*λ*	0.01	ml/ng	*k*_*cac*_ decreasing coefficient, F dependent
*E*_0_	1.38	*μ*g/l	*E* initial condition
*S*_0_	0	*μ*g/l	*S* initial condition
*L*_0_	0	*μ*g/l	*L* initial condition
*U*_0_	0	*μ*g/l	*U* initial condition
*Y*_0_	0	*μ*g/l	*Y* initial condition
*Z*_0_	0	*μ*g/l	*Z* initial condition
*F*_0_	0	ng/ml	*F* initial condition
*C*_*e*0_	1.1	cell/*μ*l×10^3^	*C*_*E*_ value at *t*=*t*_0_
*C*_*l*0_	0.3	cell/*μ*l×10^3^	*C*_*L*_ value at *t*=*t*_0_
*C*_*u*0_	0.3	cell/*μ*l×10^3^	*C*_*U*_ value at *t*=*t*_0_
*C*_*z*0_	0.3	cell/*μ*l×10^3^	*C*_*Z*_ value at *t*=*t*_0_

*E* is a generic environmental antigen: we suppose that the individual is constantly exposed to bacteria, viruses etc. and that the immune system is continuously stimulated by the corresponding exogenous antigens. When the allograft is transplanted in the recipient’s body, however, different antigen types are introduced. *S* represents antigen produced by graft cells: while these cells reproduce, this antigen is continuously formed. This antigen necessitates processing by endogenous APCs and presentation to T-cells. *S* in fact represents the non-processed form, while *L* is the processed form of the same antigen. The distinction between the processed and the non-processed forms explains the delay in processed antigen effect with respect to the directly presented antigen. *U* indicates alloantigen directly presented by donor’s APCs, which does not form anew in the organ. This antigen is ready to be presented to T-cells, so its effect in activating T-lymphocytes is rapid and strong, but vanishes as APCs are progressively cleared from the organ. Alloantigens which are indirectly presented and which derive from cells not reproducing in the allograft, have to be processed by endogenous APCs. As it was discussed in the previous section, this type of antigen does not re-form in the recipient once it is eliminated: *Y * represents the non-processed form, while *Z* represents its processed form, ready to activate T-cells. The processing mechanism is represented in the same way as for antigens *S* and *L*. The model is detailed in the following equations:

(1) $$\begin{array}{ll} \frac{\mathrm{dE}}{\mathrm{dt}}\;=\;k_{e}-k_{\mathrm{xec}}C_{e}E-k_{\mathrm{xe}}E,\;\;\;\;\;E(0)=E_{0} & \; \end{array}$$

(2) $$\begin{array}{ll} \frac{dC_{e}}{\mathrm{dt}}\;=\;k_{c}+k_{\mathrm{cac}}(F)C_{e}E-k_{\mathrm{xce}}EC_{e}-k_{\mathrm{xc}}C_{e},\;C_{e}(0)=C_{e0} & \; \end{array}$$

(3) $$\begin{array}{ll} \frac{\mathrm{dS}}{\mathrm{dt}}\;=\;k_{\mathrm{s\tau}}+k_{s}S\left(1-\frac{S}{S^{*}}\right)-k_{\mathrm{xsc}}C_{l}S,\;\;\;\;\;S(0)=0 & \; \end{array}$$

(4) $$\begin{array}{ll} \frac{\mathrm{dL}}{\mathrm{dt}}\;=\;k_{\mathrm{ls}}S-k_{\mathrm{xlc}}C_{l}L-k_{\mathrm{xl}}L,\;\;\;\;\;\;L(0)=L_{0} & \; \end{array}$$

(5) $$\begin{array}{lll} \frac{dC_{l}}{\mathrm{dt}}\;= & \;k_{c}+k_{\mathrm{cac}}(F)C_{l}L-k_{\mathrm{xcs}}SC_{l}\; & \;\\ & -k_{\mathrm{xcl}}LC_{l}-k_{\mathrm{xc}}C_{l},\;\;\;\;\;\;\;\;C_{l}(0)=C_{l0}\; \end{array}$$

(6) $$\begin{array}{ll} \frac{\mathrm{dU}}{\mathrm{dt}}\;=\;k_{\mathrm{u\tau}}-k_{\mathrm{xuc}}C_{u}U-k_{\mathrm{xu}}U,\;\;\;\;\;\;U(0)=U_{0} & \; \end{array}$$

(7) $$\begin{array}{ll} \frac{dC_{u}}{\mathrm{dt}}\;=\;k_{c}+k_{\mathrm{cac}}(F)C_{u}U-k_{\mathrm{xcu}}UC_{u}-k_{\mathrm{xc}}C_{u},\;C_{u}(0)=C_{u0} & \; \end{array}$$

(8) $$\begin{array}{ll} \frac{\mathrm{dY}}{\mathrm{dt}}\;=\;k_{\mathrm{y\tau}}-k_{\mathrm{xyc}}C_{z}Y-k_{\mathrm{xy}}Y,\;\;\;\;\;\;Y(0)=Y_{0} & \; \end{array}$$

(9) $$\begin{array}{ll} \frac{\mathrm{dZ}}{\mathrm{dt}}\;=\;k_{\mathrm{zy}}Y-k_{\mathrm{xzc}}C_{z}Z-k_{\mathrm{xz}}Z,\;\;\;\;\;Z(0)=Z_{0} & \; \end{array}$$

(10) $$\begin{array}{lll} \frac{dC_{z}}{\mathrm{dt}}\;= & \;k_{c}+k_{\mathrm{cac}}(F)C_{z}Z-k_{\mathrm{xcy}}YC_{z}\; & \;\\ & -k_{\mathrm{xcz}}ZC_{z}-k_{\mathrm{xc}}C_{z},\;\;\;\;\;C_{z}(0)=C_{z0}\; \end{array}$$

(11) $$\begin{array}{ll} \frac{\mathrm{dF}}{\mathrm{dt}}\;=\;k_{f}-k_{\mathrm{xf}}F+\frac{\kappa_{f}}{k_{\mathrm{xf}}}\delta (t-t_{\tau }),\;\;\;\;F(0)=0 & \; \end{array}$$

(12) $$\begin{array}{ll} k_{\mathrm{s\tau}}\;=\;\delta (t-t_{\tau })S_{\tau } & \; \end{array}$$

(13) $$\begin{array}{ll} k_{\mathrm{u\tau}}\;=\;\delta (t-t_{\tau })U_{\tau } & \; \end{array}$$

(14) $$\begin{array}{ll} k_{\mathrm{y\tau}}\;=\;\delta (t-t_{\tau })Y_{\tau } & \; \end{array}$$

(15) $$k_{f}=\left\{\begin{array}{c} \;0,\;\;\mathrm{if}\;\;t\leq t_{\tau }\\ \kappa_{f},\;\;\mathrm{if}\;\;t>\;t_{\tau } \end{array}\right)$$

(16) $$\begin{array}{ll} k_{\mathrm{cac}}(F)=\;k_{\mathrm{cacF}}e^{-\mathrm{\lambda F}} & \; \end{array}$$

In equation 1, describing antigen *E* dynamics, the rate *k*_*e*_ represents the entry of environmental antigens into the body, assumed to be constant throughout the considered period of time (before as well as after transplantation). The elimination terms *k*_*xec*_ and *k*_*xe*_ describe antigen neutralization due respectively to T-cell action and to T-cell-independent elimination of the antigen (as it happens *e.g.* through chemical and physical elimination mechanisms, such as lipases, mucus secreted by respiratory and gastrointestinal tracts etc.).

Equation 2 represents the dynamics of T-lymphocytes which react towards antigen *E*: *k*_*c*_ indicates constant physiological T-lymphocyte production from lymphoid organs, *k*_*cac*_(*F*)*EC*_*e*_ represents T-cell clonal expansion after antigen contact, which is inhibited by drug action, by setting the rate *k*_*cacF*_ as a proper function of the drug concentration *F*. T-lymphocytes are also “consumed” by antigen, in the sense that upon T-cell interaction with antigen, the lymphocyte eventually undergoes apoptosis (programmed cell death): this is described by the elimination term *k*_*xce*_*EC*_*e*_. We introduced another elimination term, *k*_*xc*_*C*_*e*_: lymphocytes die for apoptosis even if they do not encounter any antigen after a certain period, and we assume this mechanism to be proportional to T-cell concentration.

The regenerating antigen is described by equation 3: at time *t*_*τ*_ there is an impulsive entry, modeled by a Dirac delta (Eq. 12). Once the organ is transplanted, its cells regenerate: the growth rate is assumed to be logistic of parameter *k*_*s*_, limited by a carrying capacity *S*^∗^, so that *S* concentration tends towards *S*^∗^, whether it is above or below it. The elimination term *k*_*xsc*_*C*_*l*_*S*, depends on *S* concentration and on T-cells primed from the processed form of the antigen. The non-processed form is not ready to activate T-lymphocytes, but it is destroyed by T-cells activated from the processed form. In fact, T-cells activated from the processed antigen (*C*_*l*_) are primed to react against cells carrying the same epitopes (graft cells). Organ rejection is thus represented by the *S* antigen elimination, this being related to organ mass.

The processed form of the regenerating antigen is described by equation 4, where the only positive entry is represented by the term *k*_*ls*_*S* depending on the unprocessed antigen concentration; the two elimination terms are similar to those in the previous described equations, representing lymphocyte-dependent and -independent elimination, respectively. Equation 5 describes the *C*_*l*_ lymphocyte dynamics, with the constant entry, the clonal expansion depending on the processed antigen *L*, elimination upon encounter with both antigens *S* and *L*, and antigen-independent elimination. The directly presented antigen *U* is represented by equation 6: a Dirac’s delta describes impulsive entry at transplantation time, while elimination happens in two ways, dependent and independent from T-cells, respectively. *U*-specific T-lymphocytes, *C*_*u*_, are represented in equation 7, including constant production rate, clonal expansion, antigen-dependent and independent elimination.

The last three equations represent the non-regenerating antigen which has to be processed, its non-processed form (*Y *), the processed form (*Z*) and *Z*-specific T-cells. The dynamics are similar to the *S*, *L* and *C*_*l*_ subsystem, the only difference being that this antigen does not regenerate. Equation 8 has an impulsive entry and elimination dependent and independent from T-lymphocytes; in equation 9 the entry depends on *Y * concentration, and the usual two elimination terms follow; equation 10 is similar to equation 5. Equations 12, 13 and 14 describe the impulsive antigen entries into the system. In each case an antigen concentration (respectively *S*_*τ*_, *U*_*τ*_ and *Y*_*τ*_) is multiplied by a Dirac delta term acting at time *t*_*τ*_. The end result is the representation of the appropriate instantaneous change in *S*, *U* and *Y * compartments at the time of transplantation.

Finally, immunosuppressor pharmacokinetics is described by equation 11. For the purpose of the present model, given the long time-scale considered, drug administration is assumed to be continuous, with average rate *k*_*f*_ (equation 15) of delivery into the circulation. The drug is eliminated from the circulation following a linear, first-order process with rate constant *k*_*xf*_. Since before transplantation no treatment is administered, *k*_*f*_ is 0 before time *t*_*τ*_, while it is equal to *κ*_*f*_ from the time at which therapy begins, which we assume to be *t*_*τ*_. The term $\frac{\kappa_{f}}{k_{\mathrm{xf}}}\delta (t-t_{\tau })$ represents therefore the (impulsive) loading dose of the drug, necessary to bring it instantaneously to the equilibrium level, at which it is constant thereafter. After antigen contact, T-cells would spontaneously give rise to a (fast) clonal expansion. The immunosuppressive effect of the drug, leading to a slower increase of T-lymphocyte concentrations, is described by the exponential decrease in the clonal expansion coefficient *k*_*cacF*_ produced by proportionally increasing drug concentrations *F* (with effect rate constant *λ*): this is represented by equation 16. The drug is in fact thought to block signal transduction after antigen contact, in a concentration-dependent fashion, thus disabling clonal expansion and reducing T-cell proliferation. Equation 11 represents the pharmacokinetics of the anti-rejection drug *F* with given constant entry (depending on the administration scheme), linear elimination and with impulsive entry assumed to be simultaneous with transplantation. Equations 12-16 and Tables 1 and 2 define each symbol used. Tables 1 and 2 also report units of measurement for all variables and parameters.

### Model parameters

The model has not been fitted to experimental data, although an MLR experiment has been performed to assess the maximal lymphocyte clonal expansion rate and compute the parameter *k*_*cacF*_. Indicative parameter values for the processes modelled are difficult to find in the literature. For this reason, the main criterion followed for parameter calibration (other than *k*_*cacF*_) was the production of relative time-courses of the relevant state variables, which appeared consistent with physiology and clinical experience to the medical doctors among the authors, while remaining within a broadly acceptable range of magnitude. For instance, *k*_*xc*_ was set at 1/year, whereas values around 0.1/year can be computed indirectly from normal lymphocyte apoptosis results in vitro [18].

#### Mixed lymphocyte reaction

Blood samples were collected from four healthy donors and peripheral blood mononuclear cells (PBMCs), from three of the blood samples, were stained with the lipophilic fluorescent molecule Carboxyfluorescein Succinimidyl ester CFSE. The CellTrace CFSE Cell Proliferation Kit (Invitrogen) was used following the protocol provided by the manufacturer. Cells were stained with PBS/5%FCS/CFSE-50 *μ*M for ten minutes at room temperature and then washed twice. Non-labeled PBMCs obtained from the fourth healthy donor were used as allogenic stimuli and labeled PBMC were cultured in vitro in the presence or absence of non-labeled PBMC with a ratio of 2:1 (2×107 labeled PBMC versus 107 non-labeled PBMC). Three days later, cells were harvested and stained with PE-conjugated anti-CD3 mAb (Becton Dickinson). Cells were then analyzed at cytofluorimeter FACS-calibur (Becton Dickinson) and data were acquired by the software CellQuest pro.

#### Parameter computation

Since lymphocytes were exposed to a large amount of allogenic cells, we assume that the experiment reflects the maximal clonal expansion that can be achieved at a “maximal” antigen concentration. From equations 2, 5, 7 and 10, *k*_*cacF*_ dimensions are l/*μ*g/year. From the experiment we measured the percentage of replicating cells per day, which has to be divided by the maximal antigen concentration expressed as *μ*g/l. The concentration of MHC molecules in the experimental preparation was approximated as follows. On a cell surface there are approximately 10^5^ MHC molecules, and there were approximately 10^6^ cells/ml of blood. It thus follows that the concentration of MHC molecules was 10^5^×10^6^/ml, *i.e.*10^14^molecules per liter. Considering both MHC class I and class II molecules, the average molecular weight is approximately 60 kDa, meaning that one mole of MHC weights 60×10^3^grams. Since in one mole there is one Number of Avogadro of molecules, 10^14^ molecules correspond to 10^14^/(6×10^23^) moles, or approximately (10^18^×6)/(10^23^×6)) g/l. The parameter *k*_*cacF*_ has therefore been computed as the clonal expansion per day divided by the maximal antigen concentration (10^−5^g/l or 10 *μ*g/l), afterwards multiplied by 365 days. No formal statistical parameter estimation (*e.g.* Maximum Likelihood-based) was attempted. In keeping with the generally qualitative character of this model’s predictions, the desired outcome of the MLR experiment was an indicative, plausible value for the rate of clonal expansion, and, as is apparent, this plausible value itself is conditional on *ad-hoc* assumptions (*e.g.* that maximal stimulation is equivalent to an antigen concentration of 10 *μ*g/l).

## Results

### Clonal expansion rate from MLR

**Figure 3:**
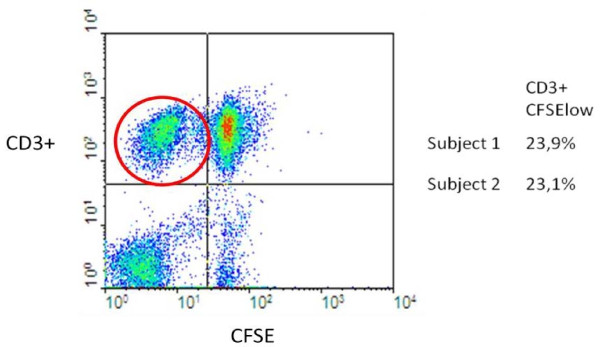
**Cytofluorimeter analysis.** Cells were analyzed for CD3 expression and CFSE incorporation. T-cells are selected with CD3+ marker, on the y axis the group of cells above the horizontal line are CD3+. On the x axis, the amount of CFSE indicates if cells replicated or not: for each cell division the amount of CFSE incorporated in DNA is reduced by half. The upper left panel (red circle) contains T-cells which have replicated.

In Figure 3 a picture from the cytofluorimetric analysis is shown. Cells labeled with anti-CD3+ are T-lymphocytes, and those who have replicated have a lower content of CFSE, thus the upper-left panel shows T-cells that have reacted to the exogenous antigen. In the two samples that replicated we had respectively 23.9 and 23.1% of cells that had divided, that is an average of 23.5%, which means that 100-23.5=76.5% of the cells did not replicate. Cells went through 3 cell cycles in 3 days, so it can be assumed that cells went through one cycle per day on average. At each cycle, cells which divide double, so the amount of cells that are allo-reactive and start replicating at day 1 is 23.5*%*/2^3^≃3*%* of the final number. So, for every 100 cells in the final count, on day 1 we have 76.5 (cells that did not replicate) + 3 (cells that replicated), that is 79.5% of the final 100%. The global replication rate at each rate was computed solving the following equation:

(17) $$y_{3}=y_{0}e^{-k_{\max}t}$$

where *y*_0_ is the total number of cells on day 0 (approximately 79) and *y*_3_ is the number of total cells on day 3 (100), while *k*_*max*_ is the replication rate. Solving (17) with *t* = 3 we obtain *k*_*max*_ = 0.08 /*day*. *k*_*cacF*_ = 0.08/10^−5^*l*/*g*/*day*, which is 2.92 *l*/*μg*/*year*.

### Model simulation

The model has been implemented in MatlabⒸ2010b, and simulations are presented showing the behavior of the several types of antigen and corresponding specific T-cell populations after transplantation of a solid organ. Three scenarios are depicted, corresponding respectively to the no-therapy, moderate therapy and maximal therapy situations. A time range from 35 to 60 years is shown, hypothesizing that transplantation occurs at time 40 years.

**Figure 4:**
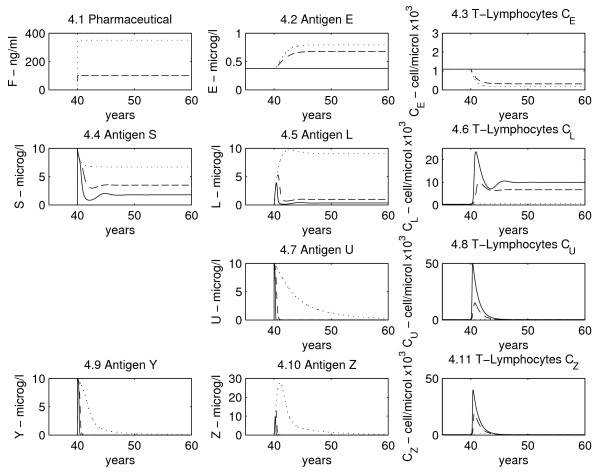
**Variables dynamics plots.** For each variable three scenarios are shown: solid line represents no therapy administration, the dashed line a middle immunosuppressant dose, and the dotted line a high dose. In Figure 4.1 the pharmaceutical dynamics is shown, while in the other plots each antigen dynamics is in line with the respective T-cell plot.

In Figure 4 all antigen types, the corresponding T-cell dynamics, and drug concentrations, are shown in the three therapy cases; subfigure 4.1 shows drug dynamics. In all subfigures the solid line (–) represents the no-therapy case, the dashed line (- -) refers to the moderate drug dose while the dotted line (.) refers to the high drug dose. Variable concentration ranges, as will be explained in the discussion, are in agreement with physiological limits.

Concentrations are assumed to be constant if no traumatic events happen during life. As the allograft is introduced (taking, *e.g.*, *t*_*τ*_ = 40 years), all antigen types described by the model (with the exception of the environmental antigen *E*) go from 0 to a high level. In response, specific T-cell concentration also grows from a normal, low level to a high one. The solid lines correspond to the no-therapy case: at the beginning antigen concentrations are very high and so T-cells are quickly activated, causing antigen elimination, which only partially regenerates (case of antigen *S* and its processed form, *L*). After this first period, as T-cells react towards the graft to destroy it, antigen concentration (which is proportional to graft mass) decreases. Following antigen reduction, T-cell concentrations are also reduced, but remain consistently higher than baseline (*i.e.* before transplantation), as antigen from the organ is continuously produced and never vanishes (T-lymphocytes *C*_*l*_, Figure 4.6). If no therapy is applied, T-cell levels remain high and will bring the organ to a minimal size (rejection and failure): as can be noticed, the continuous line in the regenerating antigen graph (concentration of antigens *S* and *L*, Figure 4.4 and 4.5) is very low.

The dashed line represents the case in which administration of a moderate amount of immunosuppressive drug (such as cyclosporine) takes place. We assume that the drug is given simultaneously with the organ transplantation and that the patient is continuously and constantly treated (subfigure 4.1). It should be noticed that, as the increase of T-cell concentration is much lower, antigen level remains higher than the no-therapy case. This indicates that the graft is not totally destroyed by the immune system.

If therapy is much stronger (dotted line), the graft normally survives. The problem is that, with immunosuppression, T-cell levels fall well below normal. As T-cells are less aggressively attacking the allograft, its regeneration allows the attainment of a constant, sizable equilibrium level, but T-cells do not proliferate as much and are thereby less effective not only towards the graft’s antigen, but also towards environmental antigens, exposing the patient to opportunistic infections. In fact, while rejection is prevented, patients might not be able to defend themselves from severe general infections. Any chosen intensity of therapy represents a compromise between desirable graft tolerance (with attending functional organ size) and dangerous lowering of general immune defenses.

SubFigures 4.2 to 4.11 show the time course of specific antigens and their respective T-cell dynamics. In subFigures 4.2 and 4.3, generic antigen and generic T-lymphocyte concentrations are reported. If no therapy is administered, these dynamics are at equilibrium. With therapy, generic T-cell concentration decreases (depending on drug dose): this indicates that immunosuppression is not specific in lowering T-cell expansion towards the graft. Instead, it reduces proliferation of all T-cells and, as a consequence, environmental antigen permanence levels in the body (*E*) increase.

In subfigures 4.4, 4.5 and 4.6, the concentrations of regenerating antigen in the non-processed (*S*) and processed (*L*) forms, as well as T-cells that respond to it (*C*_*l*_), are shown. As seen before, in the absence of therapy, low antigen and high T-cell levels are reached after transplantation, while with drug administration antigen and T-cell concentrations are respectively higher and lower. This is the only case in which antigen never goes to zero because it is produced from regenerating graft cells. Dynamics of directly presented, non-regenerating antigen (*U*) and corresponding T-cells (*C*_*u*_) are shown in subfigures 4.7 and 4.8: without therapy T-cells rapidly expand and consequently the antigen is rapidly eliminated, while, with therapy, the action of T-cells is less aggressive on this type of antigen, which will eventually be eliminated because it does not regenerate. The non-regenerating and indirectly presented antigen (subFigures 4.9 and 4.10) has to be processed: there is a delay in the increase of *Z*, which depends on non-processed antigen (*Y *) dynamics. The unprocessed antigen rapidly grows and rapidly vanishes as it is processed to the indirectly presented form and is no longer produced (because it derives from APCs not reproducing in the graft).

In subfigure 4.11 the dynamics of specific T-cells primed for antigen *Y * and *Z* is shown. In this case as well, as drug dosages increase, T-cell concentrations decrease and antigen levels rise.

From the graphs shown it is evident that is not easy to find the right drug dosage. In fact, it can be seen that when the dose is moderate T-cell levels are adequate but the graft tissue size is too small, while, as drug concentrations increase, T-lymphocyte levels are not sufficient to defend the patient from infections. The perfect situation would be to find a therapy level, which is effective in saving the graft from rejection, but does not unduly expose the individual to infections.

### Effect of immunosuppression on T-cell reaction towards infection

**Figure 5:**
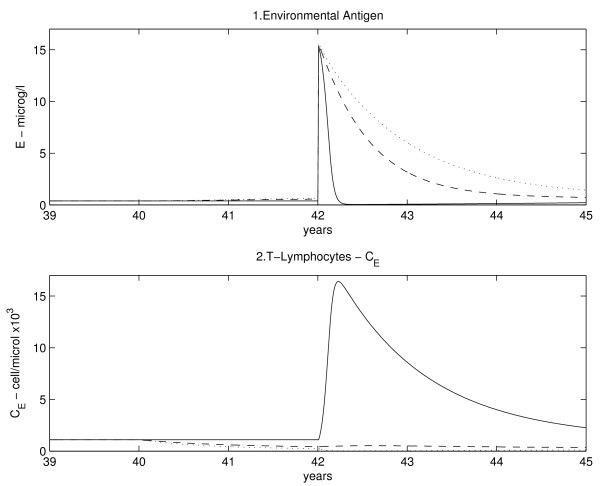
**Environmental antigen and lymphocytes reaction to infection with different drug dosages.** Solid line, no immunosuppression; dashed line, middle dose; dotted line, high dose.

In order to explore the predictive ability of the model, a simulation of an infection occurring two years after transplantation has been performed: the environmental antigen *E* and its specific T-cell population are shown in Figure 5. It is clear that, if the patient is not under treatment with the immunopuppressor (solid line) the immune system reacts normally (big increase in T-lymphocyte concentration) and the antigen is quickly brought back to equilibrium levels. If the subject is immunosuppressed, T-cell population growth is inhibited in a drug-dosage-dependent fashion, and the patient is not able to fight the infection (elevated *E* antigen levels maintained for a long time).

### Balance between organ survival and immunosuppression

**Figure 6:**
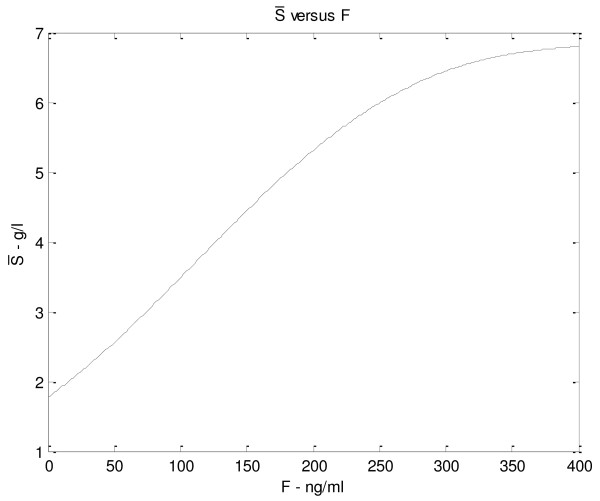
$\bar{S}$***versus F.* With increasing drug dose (x axis) the *S *carrying capacity, representing organ regeneration rate, increases and shows a saturation curve.**

**Figure 7:**
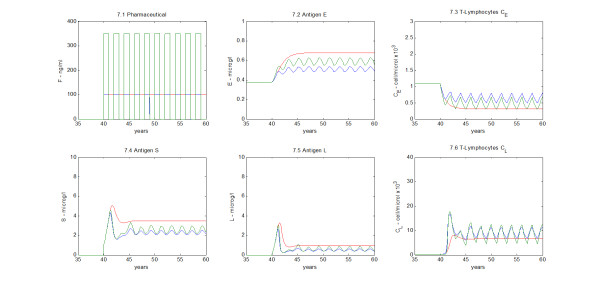
**Bifurcation diagrams of the allograft regenerating antigen indirectly presented subsystem.** The bifurcation diagrams refer to the non elementary equilibrium points of subsystem (19), according to varying amount of drug *F* (the bifurcation parameter) from 0 to 350 ng/ml.

**Figure 8:**
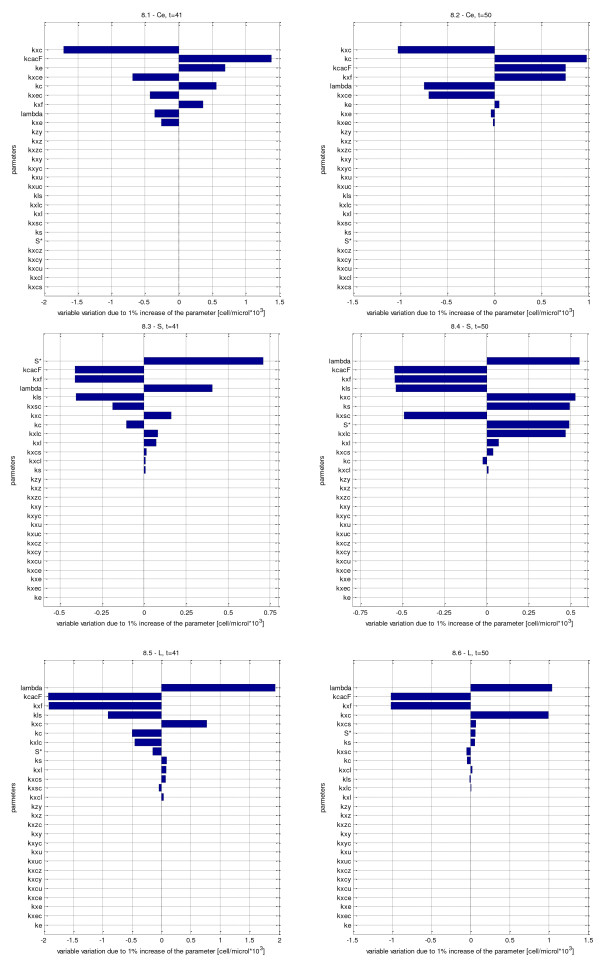
**Bifurcation diagram for *S*, the antigen produced by graft cells, non-processed form.** The bifurcation diagrams refer to the equilibrium points of *S* in (19), according to varying amount of drug *F* (the bifurcation parameter) from 0 to 350 ng/ml. The continuous line indicates asymptotically stability, the dashed line indicates instability.

**Figure 9:**
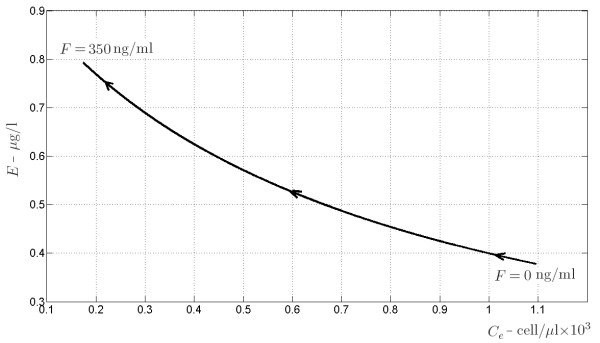
**Bifurcation diagram for *L*, the antigen produced by graft cells, processed form.** The bifurcation diagrams refer to the equilibrium points of *L* in (19), according to varying amount of drug *F* (the bifurcation parameter) from 0 to 350 ng/ml. The continuous line indicates asymptotically stability, the dashed line indicates instability.

**Figure 10:**
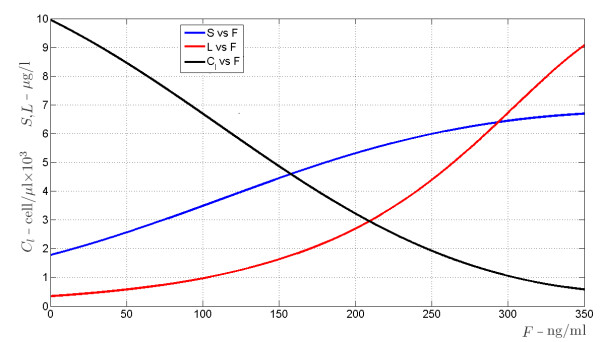
**Bifurcation diagram for *C***_***l***_, **T-cells activated from the processed antigen.** The bifurcation diagrams refer to the equilibrium points of *C*_*l*_ in (19), according to varying amount of drug *F* (the bifurcation parameter) from 0 to 350 ng/ml. The continuous line indicates asymptotically stability, the dashed line indicates instability.

As will be discussed in the Appendix, the subsystem *S*, *L* and *C*_*l*_ has two equilibrium points. One is provided by the elementary triple *S* = 0, *L* = 0, *C*_*l*_ = *k*_*c*_ / *k*_*xc*_, corresponding to a situation of no antigen *S*, which may refer to the pre-transplantation case, when the patient has no actual exogenous organ mass. According to a proper setting of the model parameters (see the Appendix for more details), this equilibrium point is shown to be stable with respect to perturbations of *L* and *C*_*l*_, and to be unstable with respect to perturbations of *S*. On the other hand, the other, asymptotically stable equilibrium point corresponds to a non-elementary solution for the three state variables and is eventually reached after transplantation, when antigen and T-cells dynamics, under a certain drug dose, are balanced. Let us denote by $\bar{S}$ the value of *S* at the stable equilibrium that is eventually reached after transplantation, which represents the carrying capacity for antigen concentration (which we assume proportional to organ mass), as explained in subsection 3.2. A simulation is performed to show how $\bar{S}$ changes in relation to varying immunosuppressant concentrations. The plot in Figure 6 shows how the value of $\bar{S}$ varies with increasing drug doses: at *F* equal 0, $\bar{S}$ has a positive (non zero) value, which is physiologically plausible as the antigen would not be completely eliminated without drug, but its concentration would be low. As drug concentrations increase, $\bar{S}$ increases following a saturation curve: at high drug levels, when lymphocytes are inhibited, the antigen equilibrium approximates its maximum level. Similar diagrams are reported in Figures 7, 8, 9, 10 (see Appendix).

### Hypothetical cyclical therapy

**Figure 11:**
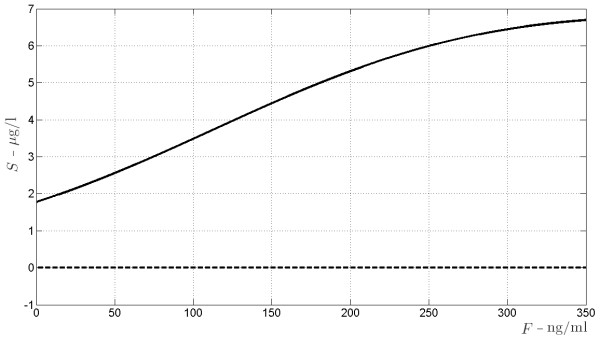
**Intermittent therapy simulation.** Red line, continuous high drug dosage; green line, high dosage cyclical therapy (every year); blue line, low dosage cyclical therapy (every year). Environmental antigen (*E*) and regenerating organ antigens (*S* and *L*) are represented, with the corresponding T-cell dynamics (*C*_*e*_and *C*_*l*_).

An additional simulation was performed to test the effect of a hypothetical “intermittent” therapy. The aim was to investigate the physiological response in the case of a cyclical administration of immunosuppressant. The period of interruption equals the period of treatment, and the given dose is the same in each treatment period. A comparison between continuous and cyclical therapies is shown in Figure 11. The time interval applied in the simulation shown is one year; several other time intervals were tested (1 week, 2, 3, 4, 6 months, 2 years) and results were similar (results not shown). As shown in Figure 11.1, the immunosuppressant is given at one year intervals. The red line represents the continuous therapy (low dose), while the blue and green lines the intermittent therapies (low and high doses). In Figures 11.2 and 11.3 the effect of the three therapy schemes on the environmental antigen (*E*) and the respective T-cells (*C*_*e*_) are shown. Figures 11.4, 11.5 and 11.6 show the dynamics of antigen *S* and its processed form *L* - representing the organ mass - and the corresponding T-lymphocytes (*C*_*l*_). The low intermittent dosage is less aggressive towards T-lymphocytes and, correspondingly, the subject is better protected against environmental antigens, but the organ is not protected from rejection as much as with the continuous dosage. The high intermittent dosage is less effective than the low continuous one, T-cells are not sufficiently inhibited and the graft antigen level is low.

### Sensitivity analysis

**Figure 12:**
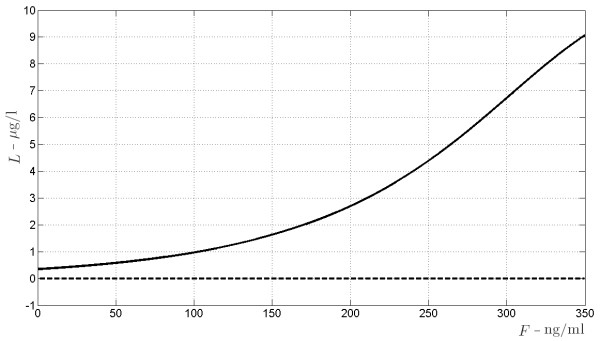
**Sensitivity analysis.** Tornado plots for selected variables (*C*_*e*_, *S* and *L*) at time 41 and 50 years. The variable percent variation for each parameter 1% increase is shown. Parameters are ordered on the y axis from the most (top) to the less (bottom) influent one.

Three variables have been identified as the most important ones to describe rejection and immunosuppression in a clinical setting: *S* and *T*, representing the organ mass (non-processed and processed form of the organ antigen) and *C*_*e*_, the T-cells specific for the environmental antigen. Sensitivity analysis of these variables at 1 year and at 10 years after transplantation (*t* = 41 and *t* = 50) has been performed (tornado plots are shown in Figure 12).

*C*_*e*_ at *t* = 41 and *Ce* at *t* = 50 plots are reported in Figures 12.1 and 12.2, respectively. The first observation is that the parameters affecting the two targets are the same, but in a different priority order. Also, at one year after transplantation, the variable *C*_*e*_ is generally more sensitive to parameter variations compared to the (near) equilibrium state at 10 years post-transplantation. The *k*_*xc*_ parameter is most influent on both variables: it represents physiological T-cell elimination, due to natural apoptosis. It is noticeable that the other elimination rate, *k*_*xce*_, which is the elimination due to T-cell interaction with the antigen, is much lower. This is understandable, since *k*_*xc*_ represents a general mechanism that involves all cells while *k*_*xce*_ only applies to cells in contact with their specific antigen. Another aspect worth noticing is that the rates which directly influence the environmental antigen (*E*), *k*_*xe*_, *k*_*xec*_ and *k*_*e*_, are important at *t* = 41 but negligible at *t* = 50 (variation 0.05% or less).

Figures 12.3 and 12.4 show tornado plots for *S* at *t* = 41 and *S* at *t* = 50. The parameters, to which the two targets for the S antigen are most sensitive, are broadly the same. The major difference is in antigen regeneration. For both *t* = 41 and *t* = 50 variations in *S*^∗^ are relevant, whereas variations in *k*_*s*_ only impact model-predicted *S* levels at 10 years post-transplantation, likely due to an accumulated effect of the small rate change. Besides the above described parameters, which regulate the *S* regeneration term, the parameters to which the variable *S* is most sensitive are the ones which regulate drug action on T-cells (*k*_*xf*_ inverse correlation with *S* increase, and lambda direct correlation with *S* increase) and the T-cell clonal expansion rate (*k*_*cacF*_). Also the transfer rate from *S* to *L* antigen forms (*k*_*ls*_) is a relevant parameter. It must be kept in mind that *S* is the non-processed form of the regenerating antigen, and it does not directly activate T-lymphocytes, while it is directly eliminated by them. In fact, the parameters governing the variation of *L*, the antigen processed form, are somewhat influent on both targets.

Tornado plots for *L* at *t* = 41 and *L* at *t* = 50 are reported in Figures 12.5 and 12.6, respectively. The processed form of the regenerating antigen is mostly affected by variations of the parameters lambda, *k*_*cacF*_ and *k*_*xf*_, as described above for the non-processed form, while it is not much affected by those parameters which regulate the processed form of *S* (*S*^∗^ and *k*_*s*_), both at time 41 and 50. One interesting observation concerns the sensitivity to the parameter *k*_*ls*_, the transfer rate from the *S* to the *L* form: one would expect an increase in this parameter to result in an increase in the target, while at *t* = 41 there is -1% variation in *L*. This is probably due to the fact that, in the moment in which the T-cell expansion stimulation is most effective, an increase in processing rate leads to an increase in the form available to activate the cells, which results in a faster clonal expansion and thus lower antigen level. At time 50, instead, the effect is almost negligible, because we are at an equilibrium situation.

As a general comment to the sensitivity analysis it is evident that the parameter *k*_*cacF*_ is surely relevant. Accurately assessing the value of the *k*_*cacF*_ parameter (derived from the MLR experiment) seems important for a correct quantitative prediction of the time course of the lymphocyte populations, as could naturally be expected. This point should be kept in mind upon applying the model in a clinical context, possibly predicting an individual patient’s post-transplantation course.

## Discussion

Mathematical models are increasingly used in biology and clinical medicine to express concise, mechanistic descriptions of ongoing phenomena. The possibility of representing a pathophysiological process by means of a mathematical model allows the investigator to formalize beliefs, compare interpretations and simulate hypothetical scenarios of interest. Mathematical models have in particular already been introduced into several areas of immunology [3-8]. So far, however, no mathematical model has yet been presented describing allograft rejection in order to support the evaluation of therapies.

The clinical problem, which characterizes the management of the transplanted patient, is the difficult adjustment of immunosuppressive therapy, walking the fine line between under-suppression, with ensuing organ rejection, and over-suppression, with the danger of potentially lethal opportunistic infections. While a wide spectrum of active pharmacological agents are now available to the transplantation specialist, their mechanism of action is often incompletely understood and their precise effect on the complex balance of immune system competence is not quantitatively determined. Therapy therefore follows rule-of-thumb principles, intensive monitoring of potential damage indicators (like serum creatinine for kidney, hepatic enzymes for liver transplantation), meticulous monitoring of plasma drug levels. The relationship between the time courses of drug effect, T-cell cycle and organ damage is however a matter of guesswork, only partially mitigated by the relatively precise knowledge of the pharmacokinetics of the immunosuppressive drugs themselves. In fact, what pharmacological information is currently offered to clinicians consists largely of single-drug pharmacokinetics parameters. Recent experiences have indeed suggested that the possibility of studying drug pharmacokinetics and pharmacodynamics through modeling techniques (*in silico*) may greatly reduce the need for animal and cellular models [19], as well as the discomfort and risks associated with extensive human experimentation. In order to work in concrete, however, the modeling approach requires a tight interconnection of mathematical constructs and physiological knowledge.

The model presented here describes established physiology mechanisms, whose outcomes, however, are not directly detectable with clinical measurements. Specific T-lymphocyte clonal expansion after foreign antigen contact, which is known to be the first step of the cascade leading to organ rejection [11], is not directly measured in clinical practice [20].

Variables concentration ranges, reported in the plots, were obtained setting the parameters in order to have a physiological reproduction of rejection, with the exception of the experimentally derived parameter *k*_*cacF*_ which was maintained constant. It is worth noticing that the concentration ranges reflect physiological values. Regarding T-cell concentrations, it has been reported that the physiological range for T-cells (CD4+ and CD8+) is 750-3600 cells/*μ*l [17], which is of course variable depending on the subject and on the situation (immune status, period of the year, general physical conditions etc.). Experiments reported in the literature give ranges of approximately 1200-1700 cells/*μ*l [21] in physiological conditions. In the plots, if we sum the concentrations of all antigen types before transplantation, we have a value of about 2∗10^3^cells/*μ*l. As regards drug concentrations, the reported values for cyclosporine treatment are 150-350 ng/ml, depending on the period following transplantation [22] which is also in agreement with our simulation: in the plot, the hypothesized drug concentrations are approximately 100 (low dose) and 350 (high dose) ng/ml. Regarding antigen concentrations, the situation is more complicated, since values for it cannot be found (as far as we know) in the literature. However, while still remaining consistent with cell-surface molecule densities as they are generally known, we may simply assume arbitrary concentrations proportional to organ mass: a rapid decrease in this arbitrary antigen concentration would signal an ongoing rejection process.

The proposed model describes the pre-transplantation equilibrium state, characterized by constant environmental antigen and T-cells level, which is dramatically perturbed by the entry of a large amount of alloantigens. Regenerating antigen determines the continuation over time of the immune system activation, leading to possible chronic rejection. The administration of therapy limits the immunological aggression towards the organ and the natural ability of the allograft to reproduce makes it so that an equilibrium is attained at a non-zero level of remaining allograft tissue. This is potentially the most useful area of application of future versions of the present model, which will incorporate, besides a general biological description of the immune response, also a precise quantification of the applicable pharmacokinetics (possibly depending on the patient or on patient subgroups).

Immunosuppressive therapy is very invasive and the substantial risks of potentially severe side effects have been widely discussed [23,24]. Models for therapy improvement (*e.g.* drug dosage) have been proposed, so far only considering single aspects of the therapy (*e.g.* plasma drug concentrations) or focusing on the action of a single specific drug [25,26]. The model presented here has instead the aim of framing drug kinetics and effects within a simplified representation of the relevant immune system biology. The somewhat empirical therapy adjustments in clinical practice, which at present are based on organ function damage indicators and drug level monitoring, may therefore be complemented, using a model similar to the one presented here, by a quantitative systemic assessment of the likely impact of posology alterations, considering therapy effects in the context of patient individual characteristics and immune system status.

The study of the present model prompts, in fact, some interesting considerations.

One aspect worth noticing, which in clinical practice is subject to iterative attempts, is therapy adjustment. The model explicitly shows that a constant dosage of one immunosuppressant is never satisfactory since no good compromise can be achieved in this way between organ survival and acceptable patient immune defenses. Even if this fact is widely appreciated among clinicians (and in fact therapy is adjusted testing it directly on the patient), there has never been, in our knowledge, a direct demonstration of it [22]. With the present model we have attempted to follow in detail the fate of several among the most meaningful cellular and chemical species involved in the immune response to organ transplantation. In so doing, we attempted a mechanistic description of those factors promoting species accumulation and decay, thereby falling naturally into the framework of mass action kinetics.

In the present work, a simulation where intermittent therapies were tested (see Figure 11) indicates that this kind of intermittent treatment would not be effective. In fact, even if the intermittent dosage is almost three times the continuous one, therapy is more effective with the latter.

In the model, the dynamics of the several types of antigen and lymphocytes are coupled through the effects of immunosuppressive therapy, acting equally on all types of T-cells. Decoupling of the several state variables, linked only by the drug effect, is actually a relevant feature of the model: therapy acts on the whole immune system; if it could act only on those cells which specifically clear graft antigens, immunosuppression would not affect individual protection against infections. This aspect also underlies how important it would be to have more specific data from transplanted patients. Also, data availability would definitely be useful for parameter identification.

Once a robust biological model is in place, it becomes relatively easy to incorporate the effect of different drugs. It will therefore be possible to express the suppression of clonal expansion, a greater mortality of T-lymphocytes, or even a generalized action in suppressing the inflammatory response (as may happen when administering corticosteroids). The problem here will not be as much in introducing the specific actions of the array of available therapy schemes, commonly used in clinical practice, but rather in representing with some degree of accuracy those side effects, which make it undesirable to simply increase without bounds the dosage of immediately useful agents. It will become possible, in this way, to support the decision-making of the attending physician or surgeon, who has to choose a reasonable compromise between immediate therapeutic effect and long-term complications.

The model presented in this work has been developed with the aim of allowing the eventual representation of different mechanisms of action, hence of the effects, of different classes of immunosuppressive drugs. Mechanisms can differ either from the molecular or the cellular viewpoint. There are different steps, along the pathway of T-lymphocyte activation, at which drugs can act (resting state, early activation, late activation and proliferation). Polyclonal anti-lymphocyte antibodies act at the resting state. Calcineurine inhibitors (cyclosporine, tacrolimus) act in the early activation pathway, so they have the same inhibition mechanism from the cellular viewpoint. However, cyclosporine and tacrolimus have different chemical structure, and act with different mechanisms at the molecular level. It has in fact been reported that tacrolimus is more effective than cyclosporine, it is used in smaller concentrations, and there are differences in their side effects [27,28]. Monoclonal antibodies and rapamycin (TOR) inhibitors act in the late activation step. Antiproliferative drugs (azathioprine and mycophenolate acid) act on the last step of the activation pathway. Corticosteroids have a very different mechanism of action in that they do not inhibit T-cell production, but they act non-specifically on the inflammatory process, preventing organ failure without directly acting on T-cell dynamics [1]. The current model can also be modified by explicitly representing different steps of the activation pathway as well as focalizing on molecular aspects for a better description of different mechanisms of action.

In this representation, some simplifications have been deliberately introduced. Among these, no discrimination has been made concerning the different T-cell types: T-lymphocytes can be either naive or activated; once activated, they differentiate into cells with specific roles (mainly helper and cytotoxic); in the present model, however, the global class of T-cells is considered, representing the response to transplantation of the immune system as a whole. The consideration of different cellular types, besides T-Lymphocytes of the CD4 and CD8 classes, would in fact be helpful in refining the description of the chain of events involved in the inflammatory response. Other cells of the immune system (*e.g.* antigen presenting cells, B-lymphocytes, macrophages etc.) as well as cytokines (responsible of cell proliferation, signaling and recruiting inflammation agents) are also involved in the rejection mechanisms. In the present work the need to limit model complexity has prompted the decision of representing only the cellular compartment (T-lymphocytes), most representative of solid organ rejection reaction, which directly increases in response to incoming antigen and triggers the rejection response. While the inflammatory response is not followed in detail, a measure of the inflammatory damage to the organ is however represented by the amount of circulating *L* antigen, assumed to be proportional to the tissue mass of living allograft.

Another simplification consists in not representing explicitly the increased overall T-cell production occurring in the presence of inflammation. When an inflammatory process is ongoing (*e.g.* during rejection) lymphoid organs are stimulated to non-specifically increase cell production. These mechanisms are poorly understood and the actual increase in competent T-cells may not be so high as to substantially modify the response: for this reason, a constant T-cell production was assumed (*k*_*c*_).

The main limitations of the current model invest both the detail of therapeutic manipulations it describes and the plausibility of the represented biology. While the model, as discussed above, can be easily and naturally extended to account for more than the single pharmaceutical agent (*F*) incorporated so far, there are in fact important aspects of the immunological response to transplantation which have not yet been tackled. One such is the description of the Graft *versus* Host Response, which is of great importance in explaining the events following transplantation of lymphoid tissue (like bone marrow transplants), particularly after massive immunosuppression of the recipient before the operation: for this reason, the present model should be considered appropriate only for solid organ transplants (liver, kidney, pancreas, heart). Another area where greater biological detail would be useful is that of the description of the chain of events in the inflammatory process which underlie the clinical features of chronic rejection. While within the framework of the present model no distinction has been made between acute (or indeed hyperacute) and chronic rejection mechanisms, factors leading to the different types of rejection may be different, and may be the object of one type of model refinement. In particular, while it is well known that acute rejection is mediated by CD4+ and CD8+ T-lymphocytes stimulated from exogenous MHC, mechanisms leading to chronic rejection are still not completely clear, and the latter is now the most common reason of graft loss from the recipient.

While the model as reported does offer useful insights in the reciprocal variations of antigen and immune cell species during a generic, hypothetical solid organ transplantation, model parameter estimation has not been carried out and no quantitative prediction can be strictly constructed, not to mention the assessment of prediction uncertainty. Indicative parameter values for the processes modeled are difficult to find in the literature. For this reason, the main criterion followed for parameter calibration was the production of relative time-courses of relevant state variables, which appeared consistent with clinical experience to the medical doctors among the authors, while remaining within a broadly acceptable range of magnitude. A priori identifiability analysis of the model has not been performed, and no data fitting has allowed us to assess a posteriori regions of confidence on parameter values. As a consequence, the model identifiability issue remains completely open.

## Conclusion

The graphs, reporting the time course of the different types of antigen and corresponding T-cell populations, show that the model captures well the clinically expected behavior of the transplanted organ mass and of the immune system reaction, under the three scenarios of no therapy, moderate therapy and aggressive therapy. From the graphs it is evident that is not easy to find the right drug dosage. It can be seen that when the drug dose is moderate T-cell levels are adequate to prevent opportunistic infections, but the *L*-antigen level, corresponding to the viable graft tissue, is rather low. Conversely, at a drug concentration effective in maintaining the entire transplanted tissue mass, the T-cell population is suppressed excessively and the risk of complications would appear to become substantial. The model therefore predicts that single drug therapy is likely to be inadequate to safely prevent graft rejection, in accord with the clinical experience so far accumulated. A perfect situation might not exist, but the theoretical exploration of drug combinations and of non-constant therapy schemes could be one way to obtain useful indications for the biological experimentation of novel therapeutic protocols.

The present work proposes then a first mathematical model of the cellular immune response to solid organ transplantation, addressing both acute and chronic rejection. The model’s mathematical behavior is broadly consistent with known physiology and long-term variations in immune status and allograft survival. The model can be tailored to address specific organ transplantation situations, may be naturally adapted to the representation of different therapeutic regimens and may offer useful indications for the optimization of therapy protocols in the transplanted patient.

## Appendix: Qualitative behavior of the solutions

From a mathematical point of view, the whole system (1-10) may be split into the four independent subsystems composing it, namely: 

• the environmental antigen subsystem:

(18) $$\begin{array}{c} \frac{\mathrm{dE}}{\mathrm{dt}}=k_{e}-k_{\mathrm{xec}}C_{e}E-k_{\mathrm{xe}}E\\ \frac{dC_{e}}{\mathrm{dt}}=k_{c}+\left(\right)k_{\mathrm{cac}}(F)-k_{\mathrm{xce}}))EC_{e}-k_{\mathrm{xc}}C_{e} \end{array}$$

• the allograft regenerating antigen indirectly presented subsystem:

(19) $$\begin{array}{c} \frac{\mathrm{dS}}{\mathrm{dt}}=k_{\mathrm{s\tau}}+k_{s}S-k_{s}\frac{S^{2}}{S^{*}}-k_{\mathrm{xsc}}C_{l}S\\ \frac{\mathrm{dL}}{\mathrm{dt}}=k_{\mathrm{ls}}S-k_{\mathrm{xlc}}C_{l}L-k_{\mathrm{xl}}L\\ \frac{dC_{l}}{\mathrm{dt}}=k_{c}+\left(\right)k_{\mathrm{cac}}(F)-k_{\mathrm{xcl}}))LC_{l}-k_{\mathrm{xcs}}SC_{l}-k_{\mathrm{xc}}C_{l} \end{array}$$

• the allograft non regenerating antigen directly presented subsystem:

(20) $$\begin{array}{c} \frac{\mathrm{dU}}{\mathrm{dt}}=k_{\mathrm{u\tau}}-k_{\mathrm{xuc}}C_{u}U-k_{\mathrm{xu}}U\\ \frac{dC_{u}}{\mathrm{dt}}=k_{c}+\left(\right)k_{\mathrm{cac}}(F)-k_{\mathrm{xcu}}))UC_{u}-k_{\mathrm{xc}}C_{u} \end{array}$$

• the allograft non regenerating antigen indirectly presented subsystem:

(21) $$\begin{array}{c} \frac{\mathrm{dY}}{\mathrm{dt}}=k_{\mathrm{y\tau}}-k_{\mathrm{xyc}}C_{z}Y-k_{\mathrm{xy}}Y\\ \frac{\mathrm{dZ}}{\mathrm{dt}}=-k_{\mathrm{xzc}}C_{z}Z-k_{\mathrm{xz}}Z+k_{\mathrm{zy}}Y\\ \frac{dC_{z}}{\mathrm{dt}}=k_{c}+\left(\right)k_{\mathrm{cac}}(F)-k_{\mathrm{xcz}}))ZC_{z}-k_{\mathrm{xc}}C_{z}-k_{\mathrm{xcy}}YC_{z} \end{array}$$

Each subsystem is driven by the common input given by the drug concentration *F*, which evolves according to a step-wise trajectory:

(22) $$F(t)=\left\{\begin{array}{c} \;\;\;\;0,\;t<t_{\tau }\\ \bar{F}=\frac{\kappa_{f}}{k_{\mathrm{xf}}},\;t\geq t_{\tau } \end{array}\right)\;k_{\mathrm{cac}}(F)=\left\{\begin{array}{c} \;\;\;\;\;k_{\mathrm{cacF}},\;t<t_{\tau }\\ k_{\mathrm{cacF}}e^{-\lambda \bar{F}},\;t\geq t_{\tau } \end{array}\right)$$

### Lemma

Each state component of the four subsystems endowed with a physiological initial condition (i.e. all positive components), admits non-negative evolutions, ∀*t* ≥ 0.

### Proof

Consider subsystem (18) and *E*(0)>0. Due to the continuity of both *E*(*t*) and *dE*/*dt*, the solution *E*(*t*) would become negative if there existed a time instant $\bar{t}>0$ such that $E(\bar{t})=0$ and ${\left(\frac{\mathrm{dE}}{\mathrm{dt}}\right|}_{t=\bar{t}}<0$, which is a contradiction because:

(23) $${\left(\frac{\mathrm{dE}}{\mathrm{dt}}\right|}_{t=\bar{t}}=k_{e}-k_{\mathrm{xec}}C_{e}(\bar{t})E(\bar{t})-k_{\mathrm{xe}}E(\bar{t})=k_{e}>0.$$

According to the same reasoning, it also readily appears that *C*_*e*_(*t*) never vanishes. The same approach can be repeated for the other three subsystems, since the delta-Dirac functions simply model a positive instantaneous increase occurring at the time *t*_*τ*_ of transplantation.•

### Lemma

As far as the environmental antigen subsystem (18) is concerned, there exists a unique positive, locally asymptotically stable equilibrium point.

### Proof

The equilibrium points of (18) satisfy the following algebraic equations:

(24) $$\begin{array}{c} k_{e}=k_{\mathrm{xec}}C_{e}E+k_{\mathrm{xe}}E\\ k_{c}+\left(\right)k_{\mathrm{cac}}(F)-k_{\mathrm{xce}}))EC_{e}=k_{\mathrm{xc}}C_{e} \end{array}$$

from which it follows that the steady state of *C*_*e*_satisfies the following second order equation:

(25) $$k_{\mathrm{xec}}k_{\mathrm{xc}}C_{e}^{2}+\left(\right)k_{\mathrm{xe}}k_{\mathrm{xc}}-k_{\mathrm{xec}}k_{c}-k_{e}\left(\right)k_{\mathrm{cac}}(F)-k_{\mathrm{xce}}))))C_{e}-k_{\mathrm{xe}}k_{c}=0$$

Since the second- and zero-order coefficients are positive and negative respectively, both solutions are real: one positive, the other negative, regardless to the sign of the first-order coefficient (see, e.g., [29]). Thus we have a unique positive solution for *C*_*e*_. As a matter of fact, by substituting the positive solution into the first equation of (24) we have a unique positive solution also for *E*:

(26) $$E=\frac{k_{e}}{k_{\mathrm{xec}}C_{e}+k_{\mathrm{xe}}}>0.$$

As for the local stability analysis, we compute the Jacobian matrix:

(27) $$J_{e}=\left[\begin{array}{c} -k_{\mathrm{xec}}C_{e}-k_{\mathrm{xe}}\;\;\;\;-k_{\mathrm{xec}}E\\ \left(\right)k_{\mathrm{cac}}(F)-k_{\mathrm{xce}}))C_{e}\;\;\;\left(\right)k_{\mathrm{cac}}(F)-k_{\mathrm{xce}}))E-k_{\mathrm{xc}} \end{array}\;\right],$$

from which the characteristic polynomial is:

(28) $$\begin{array}{c} d_{e}(\lambda )=\lambda^{2}+\left(\right)k_{\mathrm{xec}}C_{e}+k_{\mathrm{xe}}-\left(\right)k_{\mathrm{cac}}(F)-k_{\mathrm{xce}}))E+k_{\mathrm{xc}}))\lambda \\ \;\;\;+\left(\right)k_{\mathrm{xc}}-\left(\right)k_{\mathrm{cac}}(F)-k_{\mathrm{xce}}))E))k_{\mathrm{xe}}+k_{\mathrm{xc}}k_{\mathrm{xec}}C_{e}\\ \;\;=\lambda^{2}+(k_{\mathrm{xec}}C_{e}+k_{\mathrm{xe}}+k_{c}/C_{e})\lambda +k_{\mathrm{xe}}k_{c}/C_{e}+k_{\mathrm{xc}}k_{\mathrm{xec}}C_{e} \end{array}$$

Since all the coefficients are positive, the roots have negative real part [29], which means local asymptotic stability of the equilibrium point. •

### Remark

**Figure 13:**
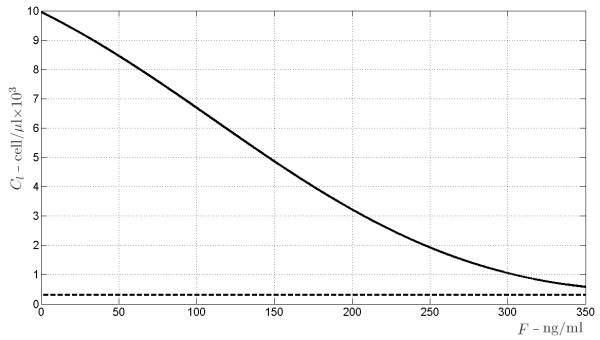
**Equilibrium points of the environmental antigen subsystem.** The equilibrium points of subsystem (18) are drawn on the (*E*,*C*_*e*_) phase plane, according to varying amount of the drug *F* from 0 to 350 ng/ml.

If we interpret the positive solution of (25) as a function of the drug $\bar{F}$ administered after transplantation it happens that, by increasing $\bar{F}$ the corresponding equilibrium for *C*_*e*_reduces its value since the first order coefficient increases, keeping unchanged the second- and zero-order terms. And, as a matter of fact, the corresponding equilibrium of *E* increases its value. These equilibria are drawn in Figure 13: increasing values of *F* go from the bottom right corner, corresponding to the pre-transplantation case *F*=0, to the upper left.

### Lemma

The allograft antigen subsystem (19) admits an elementary equilibrium point (*S* = 0,*L* = 0,*C*_*l*_ = *k*_*c*_/*k*_*xc*_), which is locally asymptotically stable if:

(29) $$k_{s}k_{\mathrm{xc}}<k_{\mathrm{xsc}}k_{c}.$$

### Proof

The existence of the equilibrium point (*S* = 0, *L* = 0, *C*_*l*_ = *k*_*c*_/*k*_*xc*_) comes out by ready computation. In order to investigate the local stability, compute the Jacobian matrix and evaluate it at the equilibrium point:

(30) $$J_{l}=\left[\begin{array}{cc} k_{s}-k_{\mathrm{xsc}}k_{c}/k_{\mathrm{xc}}\;\;\;\;\;\;0 & 0\\ \;\;\;\;k_{\mathrm{ls}}\;\;\;\;-k_{\mathrm{xlc}}k_{c}/k_{\mathrm{xc}}-k_{\mathrm{xl}} & 0\\ \;\;-k_{\mathrm{xcs}}k_{c}/k_{\mathrm{xc}}\;\;\;\left(\right)k_{\mathrm{cac}}(F)-k_{\mathrm{xcl}}))k_{c}/k_{\mathrm{xc}}\;\;-k_{\mathrm{xc}} \end{array}\;\right].$$

It clearly comes that the eigenvalues of *J*_*l*_are the elements of the diagonal: therefore, the equilibrium point is locally asymptotically stable if condition (29) is satisfied, since in that case all the eigenvalues of the Jacobian matrix are negative real. •

### Remark

It is reasonable to assume, from a physiological point of view, that the stability condition (29) is not satisfied. In this case, according to the structure of the Jacobian matrix, it happens that perturbations of the type (*S* = 0,*L* = *ε*_*L*_,*C*_*l*_ = *k*_*c*_/*k*_*xc*_ + *ε*_*Cl*_) allow a trajectory definitely convergent to the equilibrium (*S* = 0,*L* = 0,*C*_*l*_ = *k*_*c*_/*k*_*xc*_), if $\varepsilon_{L},\varepsilon_{C_{l}}$ are small enough. On the other hand, for any arbitrarily small *ε*_*S*_>0, any perturbation of the type (*S* = *ε*_*S*_,*L* = 0,*C*_*l*_ = *k*_*c*_/*k*_*xc*_) will make the trajectory diverge from equilibrium. Indeed, simulations have been carried out by setting the model parameters in order not to have inequality (29) satisfied (see Table 2).

The investigation for other equilibrium points of subsystem (29) requires the computation of the solutions of the following nonlinear algebraic system:

(31) $$\begin{array}{c} k_{s}=k_{s}\frac{S}{S^{*}}+k_{\mathrm{xsc}}C_{l}\\ k_{\mathrm{ls}}S=k_{\mathrm{xlc}}C_{l}L+k_{\mathrm{xl}}L\\ k_{c}+\left(\right)k_{\mathrm{cac}}(F)-k_{\mathrm{xcl}}))LC_{l}=k_{\mathrm{xcs}}SC_{l}+k_{\mathrm{xc}}C_{l} \end{array}$$

By making substitutions, the previous system becomes:

(32) $$\begin{array}{c} S=\frac{S^{*}(k_{s}-k_{\mathrm{xsc}}C_{l})}{k_{s}}\\ L=\frac{k_{\mathrm{ls}}S^{*}(k_{s}-k_{\mathrm{xsc}}C_{l})}{k_{s}(k_{\mathrm{xl}}+k_{\mathrm{xlc}}C_{l})}\\ \gamma_{3}C_{l}^{3}+\gamma_{2}(F)C_{l}^{2}+\gamma_{2}(F)C_{l}+\gamma_{3}=0 \end{array}$$

where:

(33) $$\begin{array}{c} \gamma_{3}=k_{\mathrm{xcs}}k_{\mathrm{xsc}}k_{\mathrm{xlc}}S^{*}>0\\ \gamma_{2}(F)=k_{\mathrm{xsc}}k_{\mathrm{xl}}k_{\mathrm{xcs}}S^{*}-k_{\mathrm{ls}}k_{\mathrm{xsc}}S^{*}\left(\right)k_{\mathrm{cac}}(F)-k_{\mathrm{xcl}}))-k_{\mathrm{xcs}}k_{s}k_{\mathrm{xlc}}S^{*}-k_{\mathrm{xc}}k_{s}k_{\mathrm{xlc}}\\ \gamma_{1}(F)=k_{s}k_{c}k_{\mathrm{xlc}}+k_{\mathrm{ls}}k_{s}S^{*}\left(\right)k_{\mathrm{cac}}(F)-k_{\mathrm{xcl}}))-k_{\mathrm{xcs}}k_{s}k_{\mathrm{xl}}S^{*}-k_{\mathrm{xc}}k_{s}k_{\mathrm{xl}}\\ \gamma_{0}=k_{s}k_{c}k_{\mathrm{xl}} \end{array}$$

A qualitative analysis would provide conditions too cumbersome to be easily treated, therefore a numerical bifurcation analysis has been carried out, according to the set of parameters reported in Table 2. The bifurcation parameter is the drug amount *F*.

It turns out that there exists a unique triple of real positive solutions (*S*,*L*,*C*_*l*_) for system (32), whose values are depicted in Figure 7 versus the drug concentration *F*. It has to be stressed that this equilibrium point is locally asymptotically stable whatever the value of *F*. Figures 8, 9, 10 refer to the bifurcation diagrams for *S*, *L* and *C*_*l*_ with respect to the drug concentration *F*.

### Lemma

There exists a unique nonnegative, locally asymptotically stable equilibrium point (*U* = 0, *C*_*u*_ = *k*_*c*_/*k*_*xc*_) for the directly presented allograft antigen (20), and there exists a unique nonnegative, locally asymptotically stable equilibrium point (*Y* = 0, *Z* = 0, *C*_*z*_ = *k*_*c*_/*k*_*xc*_) for the non-directly presented allograft antigen (21).

### Proof

The proof comes from direct computation, following the same lines of the previous Lemmas.•

## Abbreviations

APC: Antigen Presenting Cell; CNI: Calcineurine Inhibitor; MHC: Major Histocompatibility Complex; ODE: Ordinary Differential Equation; TCR: T-Cell Receptor.

## Competing interests

The authors declare that they have no competing interests.

## Author’s contributions

ADG designed the research, wrote the model equations, assessed the numerical results, and participated in writing the paper. AM participated in writing the paper, performance of the research and conducted the MLR experiment. AA participated in the performance of the research. PP participated in writing the paper and conducted the mathematical analysis. FR participated in the performance of the research. SM participated in writing of paper, in the performance of the research and assessed the clinical significance of the results. All authors read and approved the final manuscript.
